# A multivariate modeling framework to quantify immune checkpoint context-dependent stimulation on T cells

**DOI:** 10.1038/s41421-021-00352-4

**Published:** 2022-01-04

**Authors:** Léa Karpf, Coline Trichot, Lilith Faucheux, Iris Legbre, Maximilien Grandclaudon, Charlotte Lahoute, Hamid Mattoo, Benoit Pasquier, Vassili Soumelis

**Affiliations:** 1 Institut Curie, PSL University, INSERM U932, Paris, France; 2 Université de Paris, Institut de Recherche Saint-Louis, INSERM U976, Paris, France; 3 Immunology and Inflammation Therapeutic Area, Sanofi, Vitry-sur-Seine, France; 4 Université de Paris, Institut de Recherche Saint-Louis, INSERM UMR-1153, ECSTRRA Team, Paris, France; 5 Immunology and Inflammation Therapeutic Area, Sanofi, Cambridge, MA USA; 6 Assistance Publique-Hôpitaux de Paris (AP-HP), Hôpital Saint-Louis, Laboratoire d’Immunologie, Paris, France

**Keywords:** Immunology, Bioinformatics

## Abstract

Cells receive, and adjust to, various stimuli, which function as part of complex microenvironments forming their “context”. The possibility that a given context impacts the response to a given stimulus defines “context-dependency” and it explains large parts of the functional variability of physiopathological and pharmacological stimuli. Currently, there is no framework to analyze and quantify context-dependency over multiple contexts and cellular response outputs. We established an experimental system including a stimulus of interest, applied to an immune cell type in several contexts. We studied the function of OX40 ligand (OX40L) on T helper (Th) cell differentiation, in 4 molecular (Th0, Th1, Th2, and Th17) and 11 dendritic cell (DC) contexts (monocyte-derived DC and cDC2 conditions). We measured 17 Th output cytokines in 302 observations, and developed a statistical modeling strategy to quantify OX40L context-dependency. This revealed highly variable context-dependency, depending on the output cytokine and context type itself. Among molecular contexts, Th2 was the most influential on OX40L function. Among DC contexts, the DC type rather than the activating stimuli was dominant in controlling OX40L context-dependency. This work mathematically formalizes the complex determinants of OX40L functionality, and provides a unique framework to decipher and quantify the context-dependent variability of any biomolecule or drug function.

## Introduction

Context-dependency is a well-known biological phenomenon acknowledged by scientists since decades. It has been observed and described in a variety of physiological and pharmacological situations. We know that context-dependency may induce tremendous variability in the function of any cellular stimulus, biomolecule, or drug^1,2^. Thus, it explains a large part of the unpredictable tissue-, organ-, individual-, and disease-related variability in response to stimulus^3,4^.

The biological context is the environment in which cells and multicellular systems function. It is the result of a multiplicity of components that are interconnected and tightly regulated. This regulation occurs at several levels and allows cells to adjust to the situation and communicate via output signals. Cellular output responses (e.g., secretion, migration) are specific functions that have evolved to guarantee optimal cell functionality in a given microenvironment, and are constantly shaped by the combination of all contextual elements. A specific signal hitting a cell in a given context leads to an output response, but when the context composition changes, the integration of this specific signal is affected and the resulting cellular output can be qualitatively and quantitatively modulated.

Inflammation is one example of a physiopathological process that is characterized by a multitude of cellular contexts. Different immune and non-immune cell types are exposed to pro-inflammatory and anti-inflammatory signals to which they react by secreting soluble factors and by regulating the expression of surface molecules, among which are immune checkpoints (ICs). This guarantees optimal output responses with the ultimate goal of controlling the danger and maintaining tissue- and organism-level integrity. ICs play a key role in co-stimulating and co-inhibiting T cells in different situations. Most IC functions were established by studying checkpoints individually, focusing on one output function, in only one specific context. We have recently shown that CD28 harbored different functions depending on DC-expressed molecular contexts^5^. The cytokine context was shown to impact the function of OX40 ligand (OX40L) on CD4 T helper (Th) 2^6^, and T follicular helper (Tfh)^7,8^ cell differentiation. These studies contributed to a proof-of-concept for IC context-dependency. Additionally, OX40/OX40L-targeting antibodies have been used in preclinical and clinical studies in a variety of conditions, such as allergy, autoimmunity and cancer^9–11^, which are pathological situations characterized by very different types of inflammatory contexts. Hence, OX40L context-dependent functions may impact its biological role in inflammation, as well as the therapeutic response to its targeting.

Deciphering context-dependency has fundamental applications, and very broad implications in cell biology, physiopathology, and clinics. However, progress in the field has been hampered by underlying complexity^12,13^ and a formal way to analyze and quantify such a complexity is currently lacking. Dissecting the complexity of context-dependency in a global and biologically relevant manner requires a simplification of the cellular system of interest, re-creating a diversity of contexts, measuring multiple cellular outputs, and applying systematic approach to its study. To tackle this complexity, we decided to use Th differentiation as a cellular model. We set up an experimental system based on four types of parameters: (i) 15 different contexts (4 molecular and 11 cellular), (ii) one target cell type (CD4 Th), (iii) one stimulus of interest (OX40L), and (iv) a complex output response composed of 17 secreted cytokines. These four groups are of general nature and may be applied to any cell type receiving a stimulus of interest in various contexts. We then asked to what extent CD4 T cell response to the OX40L stimulus is affected by the 15 different contexts. By combining experimental systems and statistical modelling, we were able to evaluate, quantify and score OX40L context-dependency, and to specifically determine the quantitative and qualitative impact of the different contexts on the cellular output responses.

## Results

### OX40L modulates multiple Th-derived cytokines in distinct Th-polarizing contexts

**Fig. 1:**
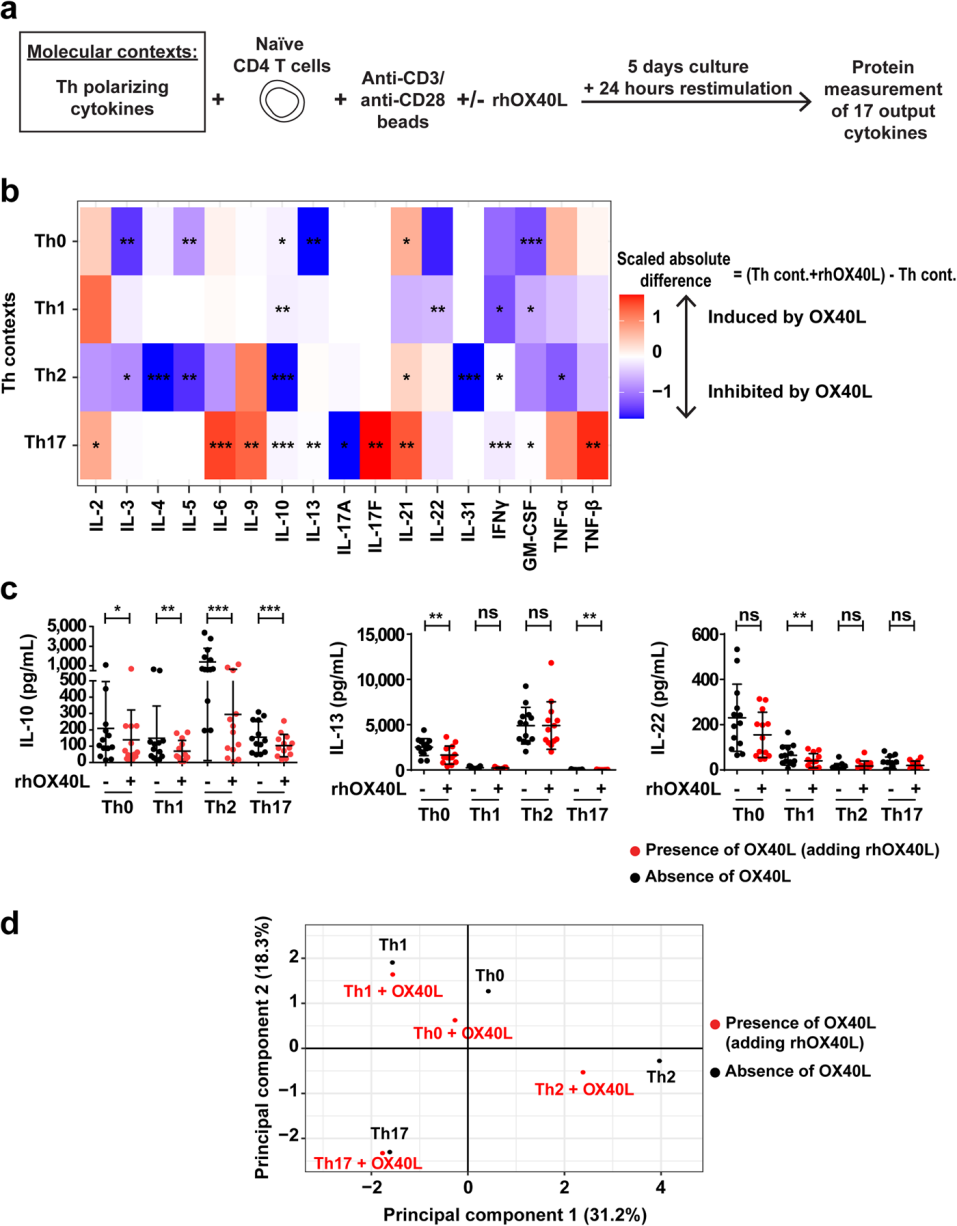
The impact of OX40L on Th cell polarization depends on the cytokine polarizing context. **a** Experimental design of the Th cell polarization assay. **b** Heatmap representing OX40L-induced scaled absolute difference on the 17 output cytokines measured in each Th context. The absolute difference corresponds to the concentration of a given cytokine in a Th context in the presence minus absence of rhOX40L. The mean over 13 donors is represented. Paired Wilcoxon’s test was used to evaluate the significance of OX40L’s effect. **c** IL-10, IL-13, and IL-22 production in each Th context in the presence (red dots) or absence (black dots) of rhOX40L. Data are the means ± SD, and individual values from 13 donors are represented. Paired Wilcoxon’s test was used for statistical analysis. **d** PCA representing the barycenters of the four Th-polarizing contexts in the presence (red dots) or absence (black dots) of rhOX40L. ns, no significance; *P ≤ 0.05; **P ≤ 0.01; ***P ≤ 0.001.

We started by studying the function of OX40L in four prototypical Th-polarizing cytokine contexts: Th0 (no added cytokine), Th1 (IL-12), Th2 (IL-4), and Th17 (IL-1β + IL-6 + IL-23 + TGF-β). Naïve CD4 T cells purified from healthy donor blood were cultured in these four contexts, in the presence of anti-CD3/anti-CD28 beads. We verified OX40 expression on T cells by flow cytometry. OX40 is not constitutively expressed on naïve CD4 T cells but is expressed as early as 24 h in culture upon TCR stimulation (Supplementary Fig. S1a). OX40 triggering was obtained by using a soluble trimeric recombinant human OX40L (rhOX40L) protein in each of the Th conditions. We assessed the Th responses after five days of culture by protein level measurement of all 17 major Th-derived cytokines (hereafter “output cytokines”) in T cell culture supernatants. This generated a dataset composed of 17 Th output cytokines in four Th contexts, from 13 independent donors (Fig. 1a; Supplementary Fig. S3, Step 1 and Data S1).

Each of the four Th contexts induced expected lineage-defining output cytokine secretion: interferon gamma (IFN-γ) for Th1; IL-4, IL-5 and IL-13 for Th2; IL-17A and IL-17F for Th17. Of the remaining output cytokines, some were detected in all conditions, such as IL-2, IL-21 and TNF-α, and others in one or two specific profiles, such as IL-31 in Th2, and IL-9 in Th2 and Th17 (Supplementary Fig. S1b).

In each of the Th contexts, we analyzed the expression of the 17 output cytokines in the presence and absence of rhOX40L. The resulting 1768 data points were represented in the form of a heatmap. We calculated absolute differences of each output cytokine concentration between the presence and absence of rhOX40L, in each Th context (Supplementary Fig. S3, Step 2). This established whether each output cytokine was induced (red), inhibited (blue), or not impacted (white) by rhOX40L (Fig. 1b). For example, rhOX40L decreased IL-10 secretion in all contexts, with the strongest effect in Th2. On the other hand, rhOX40L decreased IL-13 production in Th0 and Th17 contexts, but not in Th1 and Th2. rhOX40L also decreased IL-22 only in Th1 context (Fig. 1b, c). All individual data points for each output cytokine are shown in Supplementary Fig. S2.

Next, we addressed the impact of rhOX40L on the global Th profile, by integrating all 17 output cytokines in a principal component analysis (PCA). On the basis of the 17 output cytokines, we could clearly distinguish Th0, Th1, Th2, and Th17 conditions, as represented by their barycenter distributed in the PCA space. The presence of rhOX40L induced a shift in the global output cytokine profile, representing its functional impact. The smaller shifts were observed in Th1 and Th17 contexts, and projected along PC2 and PC1, respectively. The largest shifts occurred in Th2 along PC1, and in Th0 along both PC1 and PC2 (Fig. 1d; Supplementary Fig. S3, Step 3). Shifts induced by rhOX40L were quantitatively and qualitatively different for each context. Hence, rhOX40L induced a differential effect on output cytokine secretion according to the environment in which Th cells were differentiating, establishing a context-dependent effect of rhOX40L.

### OX40L induces a significant context-dependent effect on five output cytokines

In this first multivariate description, we noticed that OX40L context-dependent effects were also depending on the nature of the output cytokine. We developed a mathematical method to compute a score and precisely quantify context-dependency of a biological stimulus on multiple output response variables.

**Fig. 2:**
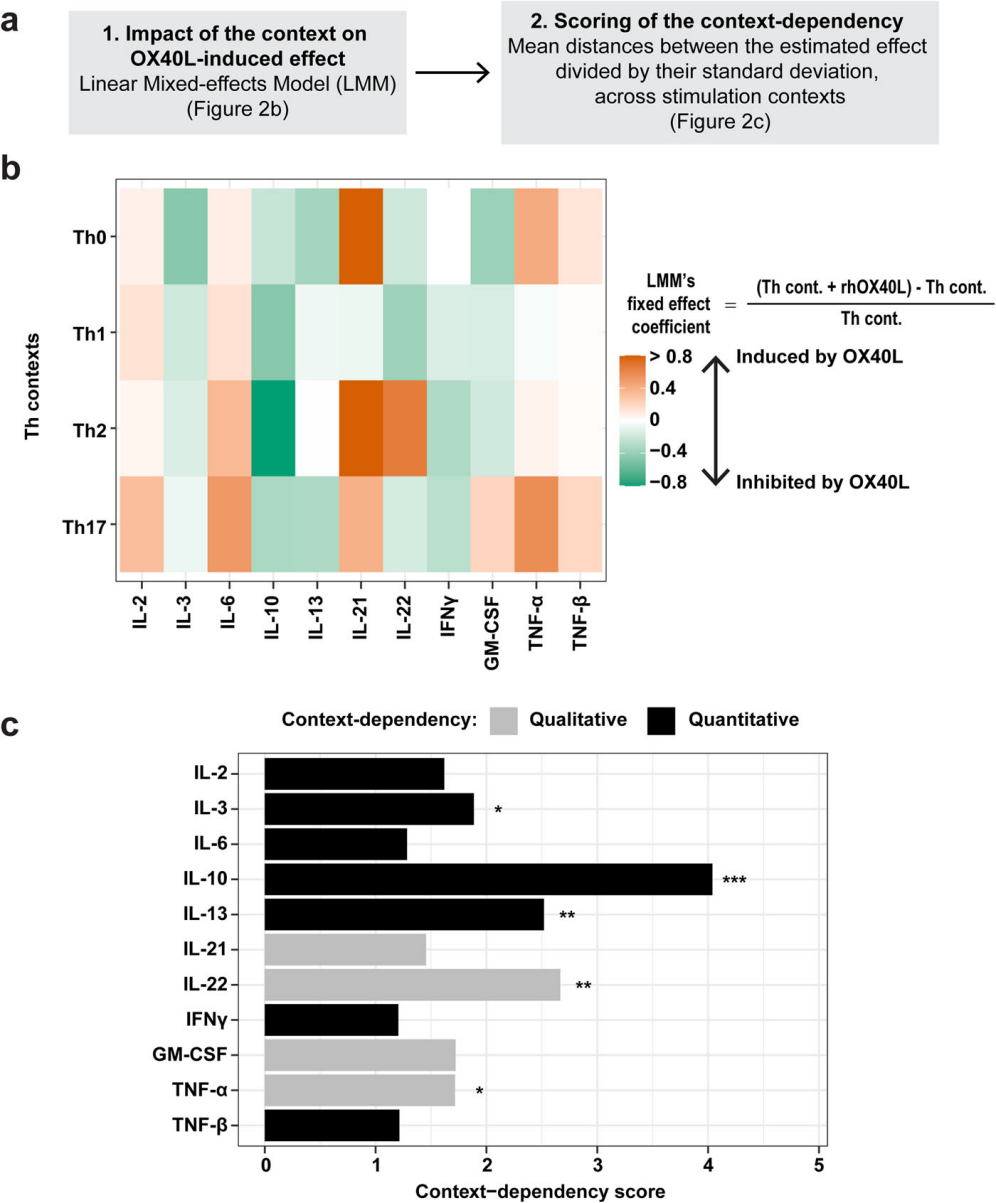
OX40L context-dependency across molecular contexts is the strongest on IL-10. **a** Simplified mathematical modeling strategy used to quantify and score the context-dependent impact of OX40L on output cytokines. In the following analyses, IL-4, IL-5, IL-9, IL-17A, IL-17F and IL-31 were excluded to avoid bias because they were produced in only one or two contexts. **b** Heatmap representing the estimated OX40L-induced relative difference for the 11 output Th-cytokines measured in each Th context, calculated using Linear Mixed-effects Models (LMM) on 13 independent donors. The estimated coefficients for the fixed effects from the models are represented. For a given cytokine, in a given Th context, the LMM’s fixed effect coefficient corresponds to the mean over donors of the concentration in the presence minus absence of rhOX40L, divided by the concentration in the absence of rhOX40L. **c** Scoring of the context-dependency across the four Th-polarizing contexts. Black bars represent a quantitative OX40L context-dependency, meaning variation of a considered output cytokine in one direction (increase or decrease) in all the Th contexts. Grey bars indicate a qualitative OX40L context-dependency, meaning variation of an output cytokine in the opposite direction in at least one Th context. The scores were computed from 13 independent donors. Likelihood ratio tests, between the LMM with and without the context variable as a fixed effect, were performed to evaluate the significance of the context-dependency. *P ≤ 0.05; **P ≤ 0.01; ***P ≤ 0.001.

In order to compare the score across the different output cytokines, we transformed the dataset to obtain OX40L-induced relative differences, taking into account donor effect and normalized concentration levels between output cytokines (Supplementary Fig. S3, Step 4.1). For each output cytokine, we applied a Linear Mixed-effects Model (LMM) (Fig. 2a; Supplementary Fig. S3, Step 4.2) to determine the effect of the context on OX40L-induced relative differences $\hat \beta$ LMM coefficient, while taking into account donor effect. Due to the specificities of our model, $\hat \beta$ can be interpreted as the mean of OX40L-induced relative differences for each context.

This method can be applied to any dataset, even if the output is not expressed in all contexts. However, we have made the choice to exclude from the analysis the cytokines that were produced only under certain conditions, such as IL-4, IL-5, IL-9 and IL-31, which are specific of the Th2 context, and IL-17A and IL-17F, which are specific of the Th17 context. In addition, they were not de novo induced in the presence of OX40L. Taking into account these output cytokines would induce a bias in the final score (because they would appear as strongly context-dependent, only due to their very narrow expression pattern), we included in the analysis only the 11 output cytokines detected at variable levels in all Th contexts to avoid this bias.

We represented the estimated OX40L-induced relative difference, $\hat \beta$, for the 11 output cytokines in each context, in the form of a heatmap (Fig. 2b). Strikingly, we observed that the effects of OX40L on a given output cytokine were highly dependent on the Th condition. We defined two types of context-dependency: (1) when OX40L was increasing or decreasing a given output cytokine concentration in all contexts but in different magnitudes (“quantitative context-dependency”), as was the case for IL-6 and IL-10 (Fig. 2b); (2) when OX40L had qualitatively different effects on a given output cytokine according to the context (“qualitative context-dependency”), as was the case for IL-22 and GM-CSF (Fig. 2b). A combination of qualitative and quantitative context-dependencies could be observed in our dataset.

Then, we computed the context-dependency score by calculating the mean distances between the different Th contexts re-scaled estimate, β’ (i.e., $\hat \beta /\hat \sigma$), to score context-dependency (Supplementary Table S1). Finally, we tested statistical significance of this context-dependency with a likelihood ratio test (Fig. 2a; Supplementary Fig. S3, Step 4.3 and Table S3).

Such statistical modeling strategy enabled to (1) directly compare and quantify OX40L context-dependency for different Th output cytokines, (2) assess the type of context-dependency, (3) assess its statistical significance (Fig. 2c). Likelihood ratio tests highlighted significant context-dependencies for five output cytokines: IL-10, IL-22, IL-13, TNF-α, and IL-3 (Fig. 2c).

OX40L decreased IL-10, IL-13 and IL-3 in all Th conditions (one-way variation: black bars in Fig. 2c), indicating quantitative context-dependency. On the contrary, IL-22 and TNF-α secretion increased or decreased in the presence of OX40L according to the Th context (two-way variation: grey bars in Fig. 2c), indicating qualitative context-dependency. This analysis of context-dependency demonstrated that (1) the molecular context (represented by distinct Th conditions) could heavily impact the function of the IC OX40L, and (2) the intensity and type of context-dependency was highly variable across the output cytokines.

This implies that a context-dependent effect for a given function may not necessarily apply to another function.

### The impact of cellular blood DC contexts on OX40L function

After molecular contexts, we wanted to apply our context-dependency score to more complex contexts, namely cellular contexts. Contrary to molecular contexts that were defined by the presence of T cell-targeting cytokines in the culture medium, cellular contexts were defined by the presence of activated DC that expressed the OX40L stimulus on their membrane. OX40L may be expressed by DC in various phenotypic and functional states, characterized by differences in multiple molecular signals delivered to T cells. We addressed the impact of such cellular contexts on OX40L functions in DC–T cell crosstalk. Immature primary BDCA1^+^ type 2 conventional blood DCs (cDC2) were sorted by fluorescence-activated cell sorting (FACS) (Supplementary Fig. S4a) from healthy donors and activated with 6 different stimuli, each defining one “cDC2 context”: Zymosan, PAM3Cys-Ser-(Lys)4 (PAM3), LPS, Heat-Killed *Staphylococcus Aureus* (HKSA), Curdlan or Thymic Stromal Lymphopoietin (TSLP). These conditions were selected for inducing distinct DC activation states^5^. Differentially activated cDC2 differed in many ways such as morphology, polarizing capacities, cytokine secretion and IC expression including OX40L expression.

**Fig. 3:**
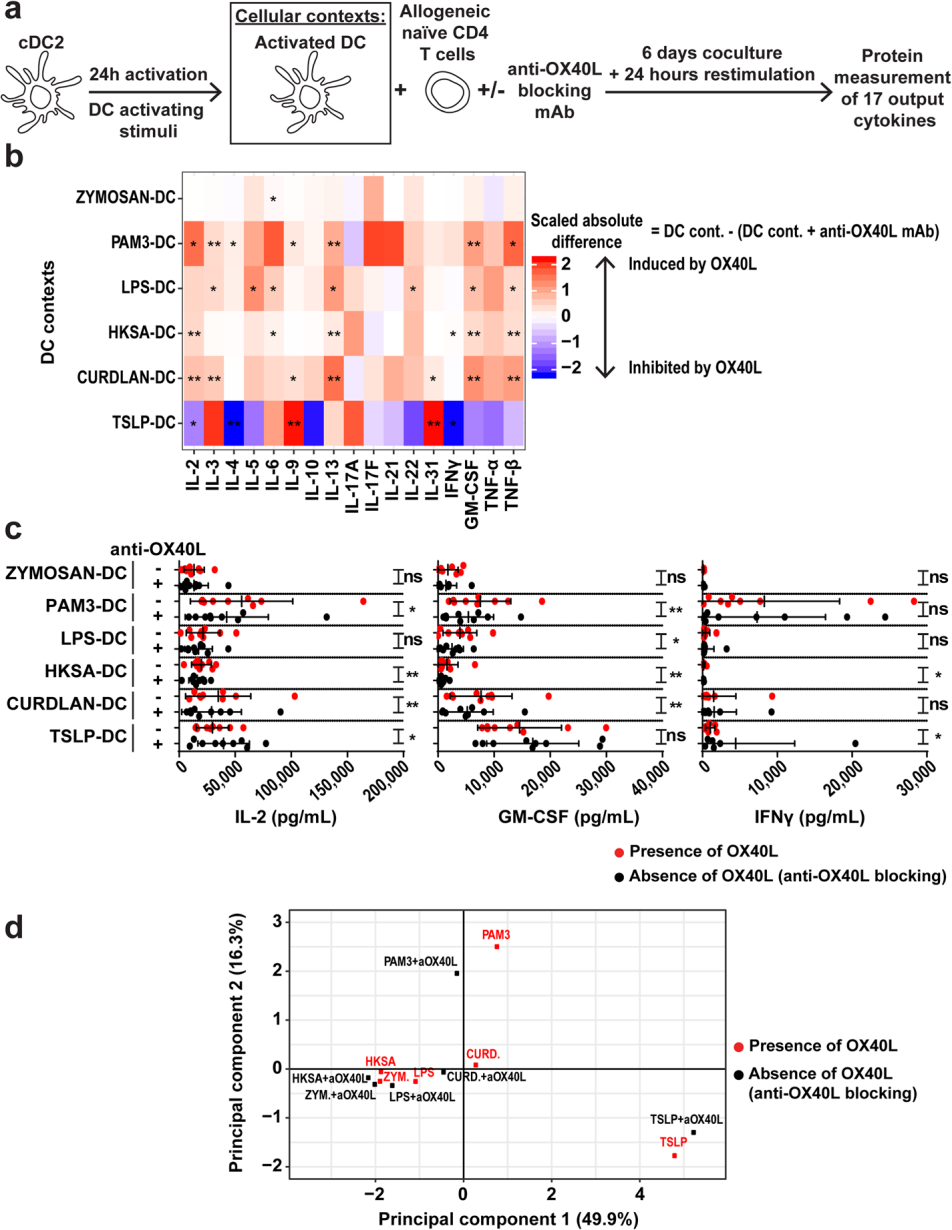
The influence of OX40L on Th cell differentiation varies depending on the cDC2-activating stimulus. **a** Experimental design of the cDC2–T coculture. **b** Heatmap representing the OX40L-induced scaled absolute difference on the 17 output Th-cytokines measured in each cDC2 context. The absolute difference corresponds to the concentration of a given cytokine in a cDC2 context in the presence of the isotype (DC cont.) minus the concentration of the cytokine in that cDC2 context in the presence of anti-OX40L antibody (DC cont. + anti-OX40L mAb). The mean is represented. Paired Wilcoxon’s test was used for statistical analysis. **c** IL-2, GM-CSF and IFN-γ production in each cDC2 context in the presence of anti-OX40L blocking antibody (black dots) or isotype (red dots). Data are the means ± SD and individual values are represented. Paired Wilcoxon’s test was used for statistical analysis. **d** PCA representing the barycenters of the six cDC2 contexts in the presence (black dots) or absence (red dots) of anti-OX40L blocking antibody, meaning absence and presence of OX40L on cDC2, respectively. Nine independent donors were used in each analysis. ns, no significance; *P ≤ 0.05; **P ≤ 0.01.

We measured OX40L expression on cDC2 after 24- and 48-h activation. OX40L was expressed in all cDC2 contexts even cDC2 cultured without stimulation (NT) (Supplementary Fig. S4b). After 24-h stimulation, activated cDC2 were cocultured with allogeneic naïve CD4 T cells in the presence of an anti-OX40L blocking antibody or matching isotype. As in the previous Th experiments, the output cytokines were measured after 6 days of coculture and 24 h of anti-CD3/CD28 restimulation (Fig. 3a; Supplementary Fig. S3, Step 1 and Data S1).

We performed the same type of analysis as in Th contexts, and calculated absolute differences in cytokine concentrations between absence (anti-OX40L antibody) and presence (isotype control) of OX40L at the DC surface, in each cDC2 context (Fig. 3b; Supplementary Fig. S3, Step 2). Results obtained for the measurements of 17 cytokines in 12 conditions for 9 donor pairs were represented in the form of a heatmap (Fig. 3b). OX40L significantly increased IL-2 in three out of six cDC2 contexts (PAM3-, HKSA-, and Curdlan-cDC2), whereas it decreased IL-2 in TSLP-cDC2 context. Similarly, OX40L increased IFN-γ in HKSA-cDC2 context and decreased it in TSLP-cDC2 context. Eventually, OX40L significantly increased GM-CSF in four out of six contexts (PAM3-, LPS-, HKSA- and Curdlan-cDC2) (Fig. 3b, c). All individual data points for each output cytokine are shown in Supplementary Fig. S5. We noticed that OX40L had a minor overall impact on output cytokines in the Zymozan-cDC2 context, as compared to the five other conditions. Strikingly, OX40L-induced effects on output cytokines in TSLP-stimulated cDC2 had a very different pattern, as compared to the five other contexts (Fig. 3b). This observation was corroborated by a PCA showing that the directional shift from “TSLP + anti-OX40L” to “TSLP” conditions was in an opposite direction when compared to the shift in the other cDC2 culture conditions. The PCA also revealed that the larger OX40L-induced shifts were observed in the PAM3-, and Curdlan-cDC2 contexts (Fig. 3d; Supplementary Fig. S3, Step 3). These analyses provided evidence of a potential OX40L context-dependency in cellular contexts.

### The TSLP-cDC2 context determines a qualitatively different OX40L-induced effect

**Fig. 4:**
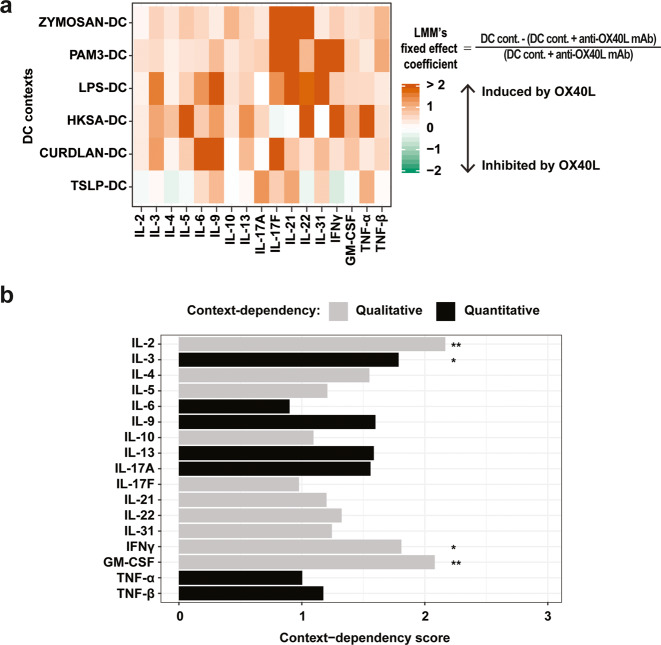
TSLP-cDC2 context determines a specific OX40L functional impact on Th output cytokines. **a** Heatmap representing the estimated OX40L-induced relative difference on the 17 output Th-cytokines measured in each cDC2 context, calculated using Linear Mixed-effects Models. The estimated coefficients for the fixed effects from the models are represented. For a given cytokine, in a given cDC2 context, the LMM’s coefficient corresponds to the mean over donors of the concentration in the presence of the isotype (DC cont.) subtracted by the concentration in the presence of anti-OX40L antibody (DC cont. + anti-OX40L mAb), divided by the concentration in the presence of anti-OX40L antibody (DC cont. + anti-OX40L mAb). **b** Scoring of the context-dependency across the six cDC2 contexts, ranked according to statistical significance. Black bars represent a quantitative OX40L context-dependency, meaning the variation of a considered output cytokine in one direction (increase or decrease) in all the cDC2 contexts in the presence of OX40L. Grey bars indicate a qualitative OX40L context-dependency, meaning variation of an output cytokine in the opposite direction in at least one cDC2 context due to the presence of OX40L. Likelihood ratio tests, between the LMM with and without the context variable as a fixed effect, were used to assess the significance of the context-dependency. *P ≤ 0.05; **P ≤ 0.01; ***P ≤ 0.001.

Next, we applied the same analysis strategy than in Th experiments to quantify OX40L-induced context-dependency in cDC2-CD4 T cell contexts (Supplementary Fig. S3, Step 4). The heatmap of $\hat \beta$ LMM coefficients (effect of the context on OX40L-induced relative differences) for the 17 output cytokines revealed different patterns of output cytokine behaviors due to OX40L presence, across the six cDC2 contexts (Fig. 4a). We found again both quantitative and qualitative context-dependencies. For example, OX40L effects on IL-6 and TNF-α were quantitatively context-dependent (one-way variations, different in magnitude), while OX40L effects on IL-22 and IFN-γ were qualitatively context-dependent (two-way variations) (Fig. 4a, b). The likelihood ratio tests highlighted significant context-dependency for four output cytokines (Fig. 4b), including IL-3 (quantitative context-dependency), GM-CSF, IL-2 and IFN-γ (qualitative context-dependency). In order to identify the conditions that were inducing these context-dependencies, we performed post-hoc likelihood ratio tests (Supplementary Figs. S3, Step 5, S4c and Table S2). Confirming the PCA observation, they indicated that these qualitative context-dependent effects were mostly due to the impact of OX40L on output cytokines in the TSLP-cDC2 context, which was very different from the others.

Thus, OX40L-induced effect on output cytokines was dependent on the cDC2 context, with a strikingly different pattern in the TSLP-stimulated cDC2.

### DC subsets differentially impact OX40L-induced effects on output cytokines

**Fig. 5:**
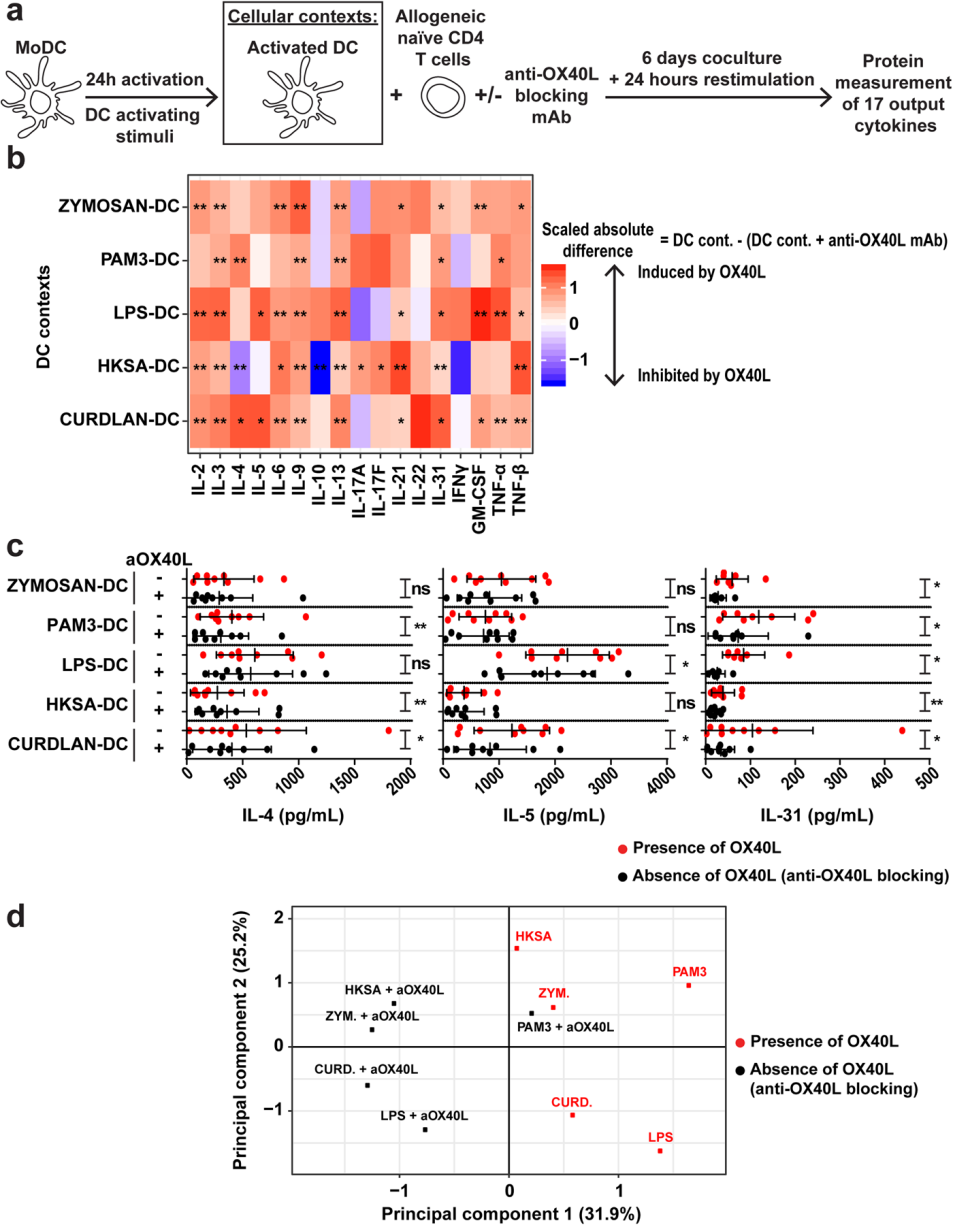
High and variable impact of OX40L on Th cell polarization in the different MoDC-activating contexts. **a** Experimental design of the MoDC–T coculture. **b** Heatmap representing the OX40L-induced scaled absolute difference for the 17 output Th-cytokines measured in each MoDC context. The absolute difference corresponds to the concentration of a given cytokine in a MoDC context in the presence of the isotype (DC cont.) subtracted by the concentration of the cytokine in that MoDC context in the presence of anti-OX40L antibody (DC cont. + anti-OX40L mAb). The mean is represented. Paired Wilcoxon’s test was used for statistical analysis. **c** IL-4, IL-5 and IL-31 production in each MoDC context in the presence of anti-OX40L blocking antibody (black dots) or isotype (red dots). Data are the means ± SD and individual values are represented. Paired Wilcoxon’s test was used for statistical analysis. **d** PCA representing the barycenters of the five MoDC contexts in the presence (black dots) or absence (red dots) of anti-OX40L blocking antibody, meaning absence and presence of OX40L on MoDC, respectively. Nine independent donors were used in each analysis. ns, no significance; *P ≤ 0.05; **P ≤ 0.01.

We have started by defining cellular contexts based on distinct stimuli given to the same DC subset. Next, we assessed whether the DC type could also determine context-dependent OX40L functions. To answer this question, we compared OX40L-induced effects on output cytokines in cDC2 versus monocyte-derived DC (MoDC) contexts, each stimulated in the same 5 conditions (Zymosan, PAM3, LPS, HKSA or Curdlan). Since TSLP receptor was not expressed on MoDC^14,15^, we did not use TSLP to activate MoDC. We verified OX40L expression on MoDC after 24- and 48-h activation in each of the MoDC culture condition. OX40L expression was significantly higher in stimulated MoDC compared to MoDC cultured without any stimulation (NT) (Supplementary Fig. S6). After 24-h stimulation, activated MoDC were cocultured with allogeneic naïve CD4 T cells in the presence of an anti-OX40L blocking antibody or corresponding isotype. The same output cytokines as before were measured in the supernatants after 6 days of coculture, and 24-h anti-CD3/CD28 restimulation (Fig. 5a; Supplementary Fig. S3, Step 1 and Data S1).

To get a global view of OX40L’s effects on output cytokine secretion, we calculated absolute differences of cytokine concentration between absence and presence of anti-OX40L, in each MoDC context (Supplementary Fig. S3, Step 2). OX40L increased IL-31 in all MoDC cultures but in different magnitude. This was also observed for IL-5, but only in LPS- and Curdlan-MoDC contexts. According to the context, OX40L induced an increase (PAM3- and Curdlan-MoDC contexts) or a decrease (HKSA-MoDC context) of IL-4 (Fig. 5b, c). All individual data points for each output cytokine are shown in Supplementary Fig. S7. PCA performed on MoDC results revealed a large shift in all MoDC contexts when comparing the presence and absence of OX40L (Fig. 5d; Supplementary Fig. S3, Step 3). Thus, OX40L-induced effects on Th output cytokines were more evenly distributed across MoDC contexts, as compared to cDC2 contexts.

**Fig. 6:**
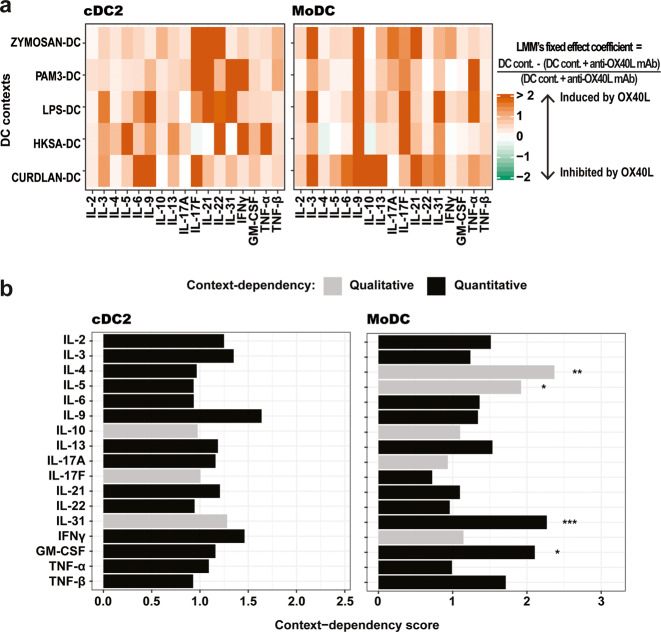
The cellular context highly impacts OX40L’s context-dependent effects on Th cytokine secretion. **a** Heatmaps representing the estimated OX40L-induced relative difference for the 17 output Th-cytokines measured in each of the five cDC2 and MoDC contexts, calculated using Linear Mixed-effects Models. The estimated coefficients for the fixed effects from the models are represented. For a given cytokine, with a given DC type and in a given DC stimulus, the LMM’s coefficient corresponds to the mean over donors of the concentration in the presence of the isotype (DC cont.) subtracted by the concentration in the presence of anti-OX40L antibody (DC cont. + anti-OX40L mAb), divided by the concentration in the presence of anti-OX40L antibody (DC cont. + anti-OX40L mAb). **b** Scoring of the context-dependency across the five cDC2 and MoDC contexts. Black bars represent a quantitative OX40L context-dependency, meaning the variation of a considered output cytokine in one direction (increase or decrease) in all the cDC2 or MoDC contexts in the presence of OX40L. Grey bars indicate a qualitative OX40L context-dependency, meaning variation of an output cytokine in the opposite direction in at least one cDC2 or MoDC context due to the presence of OX40L. Likelihood ratio tests were used to evaluate the significance of the context-dependency. *P ≤ 0.05; **P ≤ 0.01; ***P ≤ 0.001.

Next, we compared OX40L context-dependency scores for each output cytokine between cDC2 and MoDC contexts. Scores for cDC2 were re-computed without considering TSLP-cDC2 context, since MoDC were not stimulated with TSLP (Fig. 6a, b; Supplementary Fig. S3, Step 4). Interestingly, OX40L context-dependency scores showed different patterns for cDC2 and MoDC (Fig. 6b). The likelihood ratio tests highlighted significant context-dependency for four output cytokines in MoDC contexts and none in cDC2 contexts (Fig. 6b). OX40L induced an increase in IL-31 and GM-CSF concentrations in all MoDC contexts indicating a quantitative context-dependency. IL-4 and IL-5 secretion increased or decreased in the presence of OX40L according to the MoDC stimulation, indicating qualitative context-dependency (Fig. 6b). Interestingly these behaviors were not observed in the corresponding cDC2 contexts.

Thus, in addition to DC stimuli, DC type also had an impact on OX40L-induced effects on Th output cytokines. This established that the cellular context determined by either different activating stimuli, or different cell subsets, affected OX40L function.

### DC type has more impact than DC-activating stimuli on OX40L context-dependent function

**Fig. 7:**
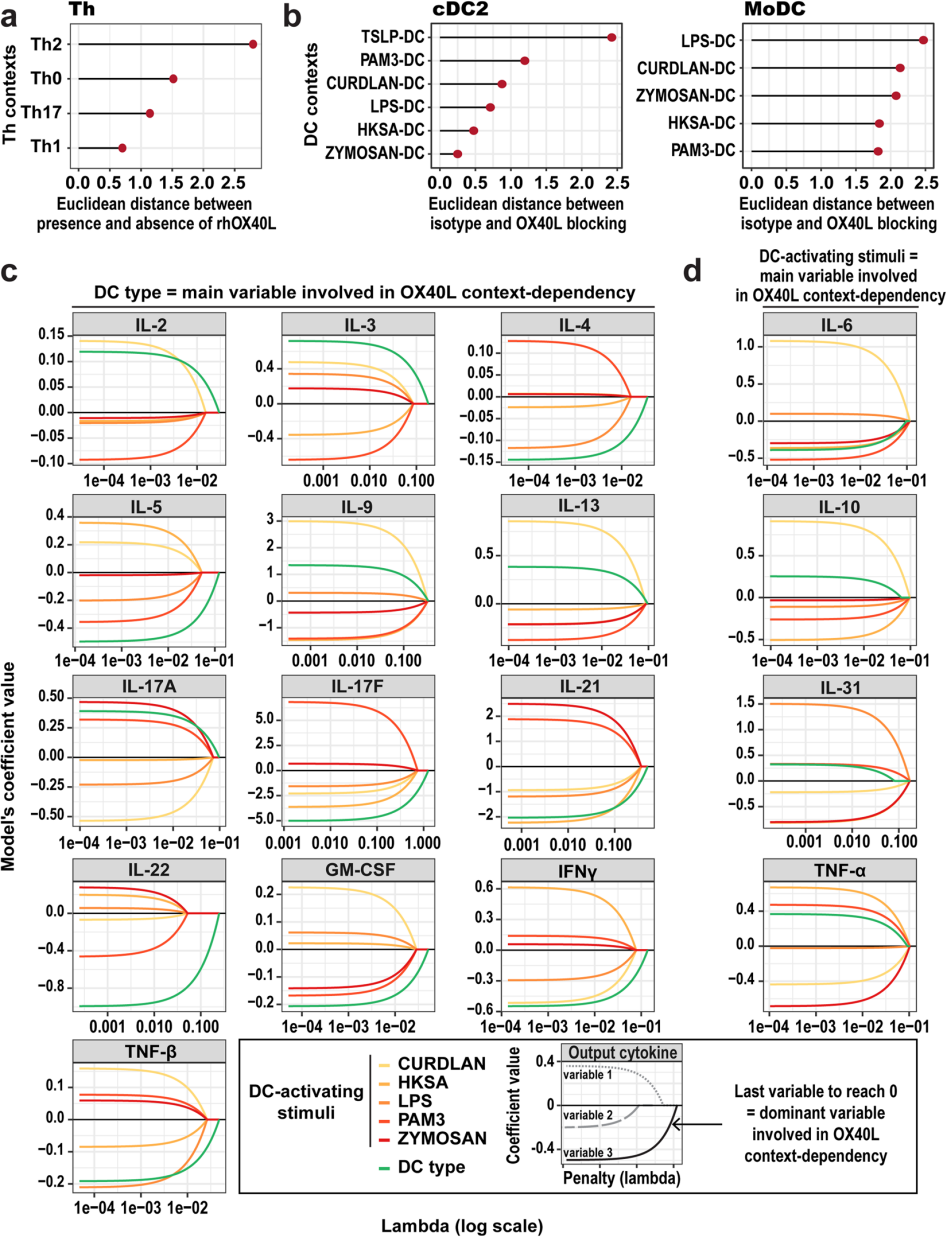
OX40L context-dependency depends mainly on the DC type. **a** Euclidean distance between presence and absence of rhOX40L for each Th context, calculated from the 17 PCs of the PCA of the raw data (all cytokines). Thirteen independent donors were used in the analysis. **b** Euclidean distance between presence (isotype) and absence (OX40L-blocking antibody) of OX40L on DCs for each cDC2 and MoDC context based on a PCA of the 17 Th-cytokines measured (17 PCs). Nine independent donors were used in the analysis. **c**, **d** For each of the 17 output Th-cytokines measured in DC–T cocultures, group-lasso coefficients as a function of the penalization parameter (lambda) are represented. **c** Cytokines for which OX40L context-dependency was predominantly due to the DC type. **d** Cytokines for which OX40L context-dependency was predominantly due to the DC contexts.

Finally, we wanted to determine which parameter had the strongest impact on OX40L-induced modulation of Th output cytokines. We compared the impact of the Th contexts, cDC2 contexts and MoDC contexts on OX40L function. Based on the previous PCA, we first calculated the Euclidean distances in each context between presence and absence of OX40L. In the Th contexts, Euclidean distance calculations revealed a higher distance between Th2 and Th2 + rhOX40L than in Th0, Th1 and Th17. This suggested that OX40L had a greater impact on output cytokines within Th2 context, followed by Th0 context (Fig. 7a). In cDC2 coculture, OX40L had a greater impact on output cytokines within TSLP-cDC2 context, as compared to the five other cDC2 contexts (Fig. 7b). Finally, in MoDC contexts, we saw fewer striking differences than in Th and cDC2 contexts. Still, LPS-MoDC context conferred higher OX40L-induced impact on output cytokines (Fig. 7b). This confirmed what we observed above, analyzing just the first two PCA components.

In addition, we went deeper in the analysis within DC contexts. In the previous analysis, we considered the DC context as cDC2 or MoDC activated with different stimuli. DC contexts were composed of two variables: the DC type and the DC-activating stimuli. Therefore, we sought to understand which of these two variables, composing DC context, revealed the strongest context-dependent effect of OX40L. To this end, for each cytokine independently, we applied a LMM to remove intra-donor correlations, followed by a group-lasso model on the residuals. We displayed the model’s coefficient values according to the lasso penalty, so that the main variable involved in OX40L context-dependency would be the last to fall to zero (Fig. 7c, d). We observed two distinct groups of cytokines, one for which OX40L context-dependency was predominantly due to the DC type (Fig. 7c), and one for which OX40L context-dependency was predominantly due to the DC-activating stimuli (Fig. 7d). For IL-9, IL-13 (DC type group), IL6, and TNF-α (DC-activating stimuli group), the penalty values required to force the coefficient to 0 for each variable were very close, therefore the interpretations about these cytokines are to consider with precaution. However, for the majority of output cytokines (13 out of 17), the DC type was the main variable involved in OX40L context-dependency (Fig. 7c). Thus, within tested cellular contexts, OX40L context-dependency on output cytokines was mostly due to the DC type.

## Discussion

Context-dependency refers to the variability in the function of a biomolecule when acting in different contexts. A major challenge in studying context-dependency lies in the tremendous diversity of possible contexts, which can be viewed at several levels: individuals, organs, sub-anatomical locations, or microenvironmental niches. Considering anatomical sites, it is known that the lung microenvironment promotes the development of Th2 responses^16^, whereas the brain promotes T regulatory (Treg) cell responses^17^. However, to our knowledge, there is no direct comparison of the function of the same biomolecule acting within lung versus brain microenvironments. This would allow to precisely determine the extent of that function, which may depend on the anatomical microenvironment. Similar questions may be asked when considering different types of inflammatory contexts: is a given molecule functioning differently when expressed in distinct types of inflammation? We and others have shown that TSLP is expressed in atopic dermatitis and activates DC to promote a Th2 response^6,15^. However, in the context of skin psoriasis, TSLP may promote IL-23 production by DC, which would favor Th17 responses^18^. This was due to the lack of IL-4, which can inhibit DC-derived IL-23^18^.

Studies at the level of an organism, an organ, or a tissue may be important to raise the possibility of context-dependent functions, but cannot be used to demonstrate and quantify context-dependency. In order to do so, a controlled experimental system is required, in which specific output functions of a given cellular stimulus would be assessed in distinct contexts. This strategy was used to establish important functional dichotomies of cytokines. For example, the differential effect of TGF-β on mouse CD4 Th cell polarization was demonstrated by comparing the absence and presence of IL-6, which promoted Treg cells and Th17 differentiation, respectively^19,20^. The importance of the cytokine context in human Th cell polarization was also established for Th17-promoting cytokines^21^, and type I interferon^13^. However, the number of contexts that has been typically studied was limited to two, and it did not allow to quantify context-dependency, nor to identify possible qualitative variations in a systematic manner. In our study, we considered 15 different molecular contexts, which necessitated the use of statistical methods in order to precisely quantify context-dependency in the form of a score. This score could be computed for various output functional markers, which enabled to rank them according to context-dependency. In our molecular contexts, this revealed that IL-10 (highest context-dependency score) was the most sensitive to OX40L context-dependency in Th-polarizing contexts, meaning that the impact of OX40L stimulus on IL-10 production was highly variable. If the inhibition of T cell-derived IL-10 would be a target of agonist or antagonistic anti-OX40L antibody, we would expect high disease-to-disease and patient-to-patient variability. Conversely, the impact of OX40L on some other T cell-derived cytokines (e.g., IL-6, IL-21, TNF-β) was very robust to the context, suggesting a more consistent effect across patients and inflammatory contexts.

Th molecular contexts also revealed previously unknown functions of OX40L, in particular the differential regulation of IL-17A versus IL-17F in the Th17 context, which has been poorly studied so far in humans^5,22–24^. Although these cytokines have similar and sometimes synergistic actions due to high homology, their role can also differ in certain contexts^25–27^. Our data provide new insights into the impact of OX40L on these Th17 cell-secreted cytokines, and could help in a better understanding of their differential regulation and secretion mechanisms.

Most studies evaluating the context-dependent effects of a stimulus have used molecularly defined conditions^6,7^. Although useful in proof-of-concept studies, using the presence and absence of a cofactor to define a biological context has several limitations. First, it creates extreme scenarios of complete absence versus high concentrations of the molecular microenvironment. This would not reflect slight variations that may be observed in physiopathology. Second, it does not reflect the complexity of inflammatory contexts, which may involve variations in contextual molecules of different nature. In our study, molecular contexts allowed us to establish a first proof-of-concept of our context-dependency score, in a highly controllable model with precise dose of polarizing cytokines, before moving to a more sophisticated model using cellular contexts. Our cellular contexts are represented by different stimulated cell types, in different activation states. It is known that distinct DC-activating stimuli are associated to very different DC molecular and functional states^5^. However, the impact of the DC state on the function of a given IC was never addressed. By studying the function of OX40L systematically across 11 DC states, we could precisely define and quantify the impact of the cellular contexts on OX40L effects in Th cell polarization.

OX40L context-dependency was very different when comparing molecular and cellular contexts. In molecular contexts, OX40L could enhance or decrease the production of an output cytokine (IL-22, TNF-α), depending on the Th-polarizing context considered. On the contrary, OX40L preferentially increased output cytokines across cellular contexts. An exception was the TSLP-cDC2 context, which influenced OX40L function in a qualitatively different manner, as compared to the other cDC2 contexts. Post-hoc tests and the analysis of cDC2 contexts without the TSLP-cDC2 dataset demonstrated that the TSLP-cDC2 condition was the most impactful on the context-dependent effect of OX40L on IL-2, IL-3, IFN-γ and GM-CSF (Supplementary Table S2). Indeed, OX40L induced a decrease of IL-2, GM-CSF, and IFN-γ, in the TSLP-cDC2 context, while inducing an increase of the same cytokines in the five other contexts. IL-2 and IFN-γ are often defined as Th1 cytokines. GM-CSF can be secreted by several Th cell subsets, and is known to stimulate the proliferation and activation of myeloid cells, and to enhance phagocytosis, antigen presentation and proinflammatory cytokine secretion^28^. This could be explained by the fact that Zymozan-, PAM3-, LPS-, HKSA- and Curdlan-cDC2 contexts are mimicking an external pathogen, of fungal or bacterial origin, while TSLP is acting as an inflammatory cytokine produced by epithelial cells or stromal cells, in the context of allergy. The increase of these three cytokines by OX40L in fungal/bacterial infection contexts, and conversely their decrease in allergic contexts seem to be consistent with the required immune response in these distinct inflammatory conditions. This emphasizes the peculiarity of the TSLP-cDC2 context, and shows that the function of an IC cannot be dissociated from the state of the DC expressing it.

The fact that TSLP-cDC2 context seems to be the main cause of OX40L context-dependency, may suggest that Zymozan-, PAM3-, LPS-, HKSA- and Curdlan-cDC2 are contexts in which OX40L will always have the same function on output secreted cytokines. However, context-dependency scores obtained in MoDC contexts challenged this idea. Context-dependency scores were calculated only across Zymozan-, PAM3-, LPS-, HKSA- and Curdlan-MoDC contexts because MoDC do not respond to TSLP due to the lack of TSLP receptor expression^14,15^. In contrast to cDC2 results, context-dependency scores for certain cytokines, such as IL-4, IL-5, IL-31 and GM-CSF, were significant. This was the first evidence that the DC-activating stimulus may not be the only parameter influencing OX40L context-dependent functions. The use of group-lasso models allowed us to identify the respective contribution of DC type versus DC-activating stimuli in the control of OX40L function. This revealed that the DC type was the most influential variable. This should encourage future studies of cell-derived biological stimuli to consider the cell type as a potential key determinant of functional variability.

A “context-independent” behaviour of OX40L on output cytokines could also be interesting to define. For instance, we observed no context-dependency of OX40L function on IL-21, neither in molecular nor cellular contexts. OX40L always increased IL-21 cytokine secretion by CD4 T cells. The positive link between OX40L, IL-21 and Tfh cells has been well-established in human in vitro assays using CD4 T cells activation by CD3/CD28 beads^7^ and by TSLP-activated cDC2^8^. Our model further supports a robust physiopathological connection between OX40L on DC, and IL-21 secretion by Th cells.

The large variability of molecular and tissue contexts during inflammation raises the question of how accurately they may be represented and mimicked in experimental systems. In our study, we considered 4 molecular and 11 DC contexts, as defined by the combination of DC type and activating stimulus. This represents a large diversity of contexts, with broad physiopathological relevance to bacterial (LPS, HKSA, Curdlan) and fungal (Zymosan) infections, as well as allergy (TSLP). However, different inflammatory conditions may induce diverse DC states. In cancer, the microenvironment induces peculiar DC programs, which may either promote or control tumor development^29^. Such complex tissue microenvironments are difficult to recapitulate in controlled systems, and require dedicated studies. The availability of data resources on tumor DC states^30,31^, and biological resources from human tumors, should facilitate the generation of tumor “DC contexts” for systematic studies. Our results suggest that distinct DC contexts may influence the function of various ICs, and possibly modify the efficiency of IC-targeting strategies.

OX40 has been considered as a drug target to enhance or inhibit T cell responses for over 20 years^32^, in a number of pathological conditions, spanning from allergy and autoimmune diseases to cancer, which are characterized by extremely different inflammatory conditions^11,33,34^. OX40 blockade demonstrated promising effects in mouse models of rheumatoid arthritis^10,35^. Interestingly, OX40 agonists used in tumor models increased mice survival and tumor-specific CD4 memory T cells in a cancer type- and anatomical site-dependent manner, suggesting that the context could significantly influence treatment efficiency^36,37^. More recently, OX40 agonists associated with PD-1 blockade increased antitumor immunity in a transplanted mouse mammary tumor model^38^. In human clinical trials, anti-OX40 monoclonal antagonist antibody has shown efficacy in atopic dermatitis^39,40^. However, OX40 agonist targeting in cancer has yet to demonstrate an effectiveness, although promising results were obtained in early-stage studies^11,41^. Variable patient response to OX40 agonists echoes previous studies showing contextual effects in mouse models, and in human Th polarization assays with a limited number of contexts^6,7^. Our study establishes the context-dependency of a controlled stimulus, OX40L, in diverse molecular and cellular contexts. Most importantly, it enables to precisely quantify the underlying determinants of context-dependent activities. The systematic application of our conceptual and methodological framework to the study of other cell stimuli, biomolecules, and drugs, will allow to score and rank their context-dependency and to identify their most contextual functional outputs. Any molecular function can be dissected in contexts associated with a given physiopathological situation. In pre-clinical drug assessment, our context-dependency score could help choosing between several molecules targeting the same pathway, by selecting the lowest context-dependent agent on the parameters that are the most relevant to those specific clinical settings. To the same end, combinatorial therapies appear to be an attractive solution due to the limited number of patients responding to IC targeting in cancer therapy, and our score could also guide drug combinations. Moreover, it often happens that molecules that have been developed for a given pathological condition are repurposed for a different disease, without having a strong biological rationale behind this choice. A context-dependency score could be used here to evaluate and quantify the context-dependency of this pharmacological manipulation of a given pathway in different disease contexts.

Together, such new knowledge could help in a rational selection of molecules to target in a given inflammatory condition that is unique to each disease, and to identify the determinants of drug response variability in individual patients. On the contrary, ignoring major context-dependent effects may create an obstacle to the efficient targeting of highly contextual molecules and pathways.

## Materials and methods

### PBMC purification

Fresh apheresis blood from healthy human blood donors was obtained from Etablissement Français du Sang (French Blood Establishment) after written informed consent, under an ethically-approved convention with Institut Curie and INSERM, according to national regulations. Peripheral blood mononuclear cells (PBMCs) were isolated by centrifugation on a density gradient (Lymphoprep, Proteogenix).

### Naïve CD4 T cell purification

Naïve CD4 T cells were purified from PBMCs using the EasySep™ Human Naïve CD4 T Cell Isolation Kit (StemCell Technologies) to reach 95% purity as CD4^+^CD45RA^+^CD45RO^–^ cells.

### Cytokine contexts for Th cell polarization

Naïve CD4 T cells were cultured for 5 days in X-VIVO^TM^ 15 medium (Lonza) with only anti-CD3/anti-CD28 Dynabeads (Life Technologies) to obtain Th0, or in combination with either 10 ng/mL IL-12 (R&D Systems) to obtain Th1, 25 ng/mL IL-4 (R&D Systems) to obtain Th2, or a cocktail of 100 ng/mL IL-23 (R&D Systems), 10 ng/mL IL-1β, 1 ng/mL TGF-β and 20 ng/mL IL-6 (Peprotech) to obtain Th17 as already published^6^. When indicated, 600 ng/mL rhOX40L (R&D Systems) was added to the T cell culture. At the end of the culture T cells were washed, counted and reseeded at 10^6^ cells/mL and restimulated with anti-CD3/CD28 Dynabeads (Life Technologies) for 24 h before collecting supernatants for cytokine measurement.

### Blood dendritic cell purification

Blood dendritic cells were purified using the EasySep Human Myeloid-DC Enrichment Kit (Stem Cell Technologies). cDC2 were sorted on a MoFlo Astrios sorter (Beckman Coulter) to reach 98% purity as Lineage (CD3, CD14, CD16, CD19, CD20, CD56)^–^, CD4^+^ (BD), CD11c^+^ (Biolegend), BDCA1^+^ (ThermoFisher), BDCA3^–^ (Miltenyi Biotec).

### MoDC generation

CD14^+^ cells were selected from PBMCs using magnetically labeled anti-CD14 Microbeads and MACS LS columns following the manufacturer’s instructions (MiltenyiBiotec). CD14^+^ cells were then cultured with IL-4 (50 ng/mL) and GM-CSF (10 ng/mL) (MiltenyiBiotec) for 5 days in RPMI 1640 Medium, GlutaMAX (Life Technologies) with 10% Fetal Calf Serum.

### cDC2 and MoDC activation

Sorted cDC2 and MoDC were seeded at 10^6^ cells/mL in a flat bottom 96-well plate and activated for 24 h using 10 µg/mL PAM3CSK4 (Invivogen), MOI 10 HKSA (Invivogen), 10 µg/mL Zymosan (Sigma-Aldrich), 10 µg/mL Curdlan (Invivogen) or 100 ng/mL LPS (Invivogen) in RPMI 1640 Medium, GlutaMAX (Life Technologies) with 10% Fetal Calf Serum (Hyclone) 100 U/mL Penicillin/Streptomycin (Gibco), MEM Non-Essential Amino Acids (Gibco) and 1 mM Sodium Pyruvate (Gibco). cDC2 were also activated using 50 ng/mL TSLP (R&D Systems).

### DC/T coculture

After 24 h activation, cDC2 and MoDC were counted and cocultured with allogeneic naïve CD4 T cells, at a ratio of 1 DC for 5 T cells, in serum-free X-VIVO 15 medium (Lonza). When indicated, 10 µg/mL anti-human OX40L monoclonal antibody (Oxelumab, Absolute Antibody) or matching human IgG1 isotype were added to the coculture and maintained for the whole duration of the coculture. After 6 days of coculture, T cells were washed and live cells were counted. T cells were reseeded at 10^6^ cells/mL and restimulated with anti-CD3/CD28 Dynabeads (LifeTechnologies). Twenty-four hours later, supernatants were collected to measure the T cell cytokines.

### Flow cytometry analysis

For surface flow cytometry analysis, dead cells were first stained using Live/dead fixable yellow dead cell stain kit (Thermo Fisher). T cells were stained with an antibody recognizing OX40 (Biolegend), while MoDC and cDC2 were stained with an antibody recognizing OX40L (BD). Cells were acquired on a ZE5 instrument (BioRad).

### Cohorts’ description

For the cytokine-induced Th cell polarization experiments, 13 independent donors were included, in five independent experiments.

In each cDC2–T cell and MoDC–T cell coculture experiment, one single cDC2 or MoDC donor was coupled to a different naïve CD4 T cell donor. For the MoDC–T coculture experiments, nine independent MoDC donors were included, and cocultured with nine different T cell donors, in three independent experiments. For the cDC2–T coculture experiments, nine independent cDC2 donors were included, and cocultured with nine different T cell donors in six independent experiments. The nine TSLP-cDC2 donors were processed in different experiments than the nine donors of the other cDC2 contexts, so the donors are different from the other cDC2 contexts.

### Statistical analysis

All concentration values below the limit of detection (LOD) of 10 pg/mL were set to LOD/2 (5 pg/mL). Luminex measurements (four cytokines) were realized later than CBA measurements (13 cytokines). Because of the limited amount of material for some donors, we prioritized measurements of the output cytokines by CBA over Luminex. Supernatants from 12 samples (out of 108 samples) of the cDC2–T coculture experiments and from 10 samples (out of 90 samples) of the MoDC–T coculture experiments were missing and measurement of four cytokines (IL-21, IL-22, IL-31, and TNF-β) could not be performed. To handle missing values, stochastic single imputation was performed using Multiple Imputation by Chained Equations (MICE). Absolute and relative differences in concentration induced by OX40L were computed. Two-sided paired Wilcoxon tests were used to compare raw concentrations and absolute differences across stimulation contexts; mean and standard deviation are displayed.

### Context-dependency score

The following steps were used to compute the context-dependency score, for each output cytokine independently: (i) using the relative differences in concentration induced by OX40L, the estimated effect for each stimulation context ($\hat \beta _{context\;k}$) and its standard deviation ($\hat \sigma _{context\;k}$) were obtained from a LMM featuring the different contexts as fixed effects and donor-specific intercepts as random effects (Eq. (1));

1 $$Y = {{{{\alpha }}}}_{{{{{donor}}}}} + \mathop {\sum }\limits_{{{{\mathrm{context}}}}\;k} \beta _k1_k + {{{{\varepsilon }}}}$$

where $Y$ is the relative difference in concentration induced by OX40L presence for a given cytokine; 1 is the indicator function, ${{{{\beta }}}}_{{{{k}}}}$ is the fixed effect for stimulation context *k*; ${{{{\alpha }}}}_{{{{{donor}}}}}$ is the random intercept associated to each donor, with $\alpha _{donor} \sim {{{\mathcal{N}}}}(0,{\upnu}^2)$; and $\varepsilon \sim {{{\mathcal{N}}}}(0,\sigma ^2)$ is the residual error term (Supplementary Fig. S3, Step 4.2); (ii) Pairwise Euclidean distances between the context-specific estimated effect divided by their standard deviation $\left( {\frac{{\hat \beta _{context\;k}}}{{\hat \sigma _{context\;k}}}} \right)$ were computed (Supplementary Fig. S3, Step 4.3); (iii) Last, the context-dependency score was obtained by taking the mean of the pairwise Euclidean distances. A likelihood ratio test, between the LMM with and without the context variable as a fixed effect, was performed to evaluate the significance of the context-dependency (Supplementary Fig. S3, Step 4.3). The likelihood ratio test addressed a different question from absolute differences. In a few words, this test assessed the goodness-of-fit of two models, one that did not take into account the contexts and one that took into account the distinct contexts, and evaluate which is better supported by the data.

The scores were then represented according to the OX40L-induced effect on the different contexts: when OX40L effect was in the same direction for all contexts (output cytokine production either always induced or always inhibited), the score was represented with a black bar (quantitative context-dependency). However, when OX40L effect went in both directions (inducing or inhibiting the output cytokine production, depending on the context), the score was represented with a grey bar (qualitative context-dependency).

To evaluate the difference in OX40L effect between pairs of contexts, likelihood ratio tests, between the LMM with and without the pair of context variable as a fixed effect, were performed (only observations for the two contexts were used).

Relative importance of DC type and the DC-activating stimuli on the context-dependent effect of OX40L was evaluated with a two-step method, for the relative difference of each cytokine independently: (i) first a LMM including only an intercept as fixed and random effect was fitted; (ii) the residuals from this model were used as the response in a group-lasso model. All stimulation contexts were defined as one group in the lasso model in order to compare the two types of context: DC type and activating stimuli.

### Cytokine quantification

Cytokines from T cell supernatants were quantified using CBA flex set for IL-2, IL-3, IL-4, IL-5, IL-6, IL-9, IL-10, IL-13, IL-17A, IL-17F, TNF-α, IFN-γ and GM-CSF (BD), and Luminex for IL-21, IL-22, IL-31 and TNF-β following the manufacturer’s protocol.

## Supplementary information

Supplementary Figures

Supplementary Table S1

Supplementary Table S2

Supplementary Table S3

Supplementary Data S1

Supplementary Data S2

Supplementary Data S3

## Data Availability

All data generated and analyzed during this study are included in Supplementary Data S1. It contains all concentrations of the 17 Th output cytokines in the presence or absence of OX40L in Th, cDC2 and MoDC contexts (for independent donors).

## Software and code availability

FACS data were analyzed using the FlowJo software (TreeStar).

Software used for CBA analysis was FCAP Array v3.

Statistical analysis was performed using the Prism software (GraphPad) and R software (version 3.5.3).

Code to reproduce the results is available in Supplementary Data S2 (OX40L_ContextDependency_ThContexts.R) and Supplementary Data S3 (OX40L_ContextDependency_DCcontexts.R).
