# Supplementary material for: A multivariate modeling framework to quantify immune checkpoint context-dependent stimulation on T cells
Source: Cell Discov. 2022 Jan 4;8:1. doi: 10.1038/s41421-021-00352-4 (PMC8727669; doi:10.1038/s41421-021-00352-4)
Supplement: Supplementary file 1 — Supplementary Figures [file 41421_2021_352_MOESM1_ESM.pdf]

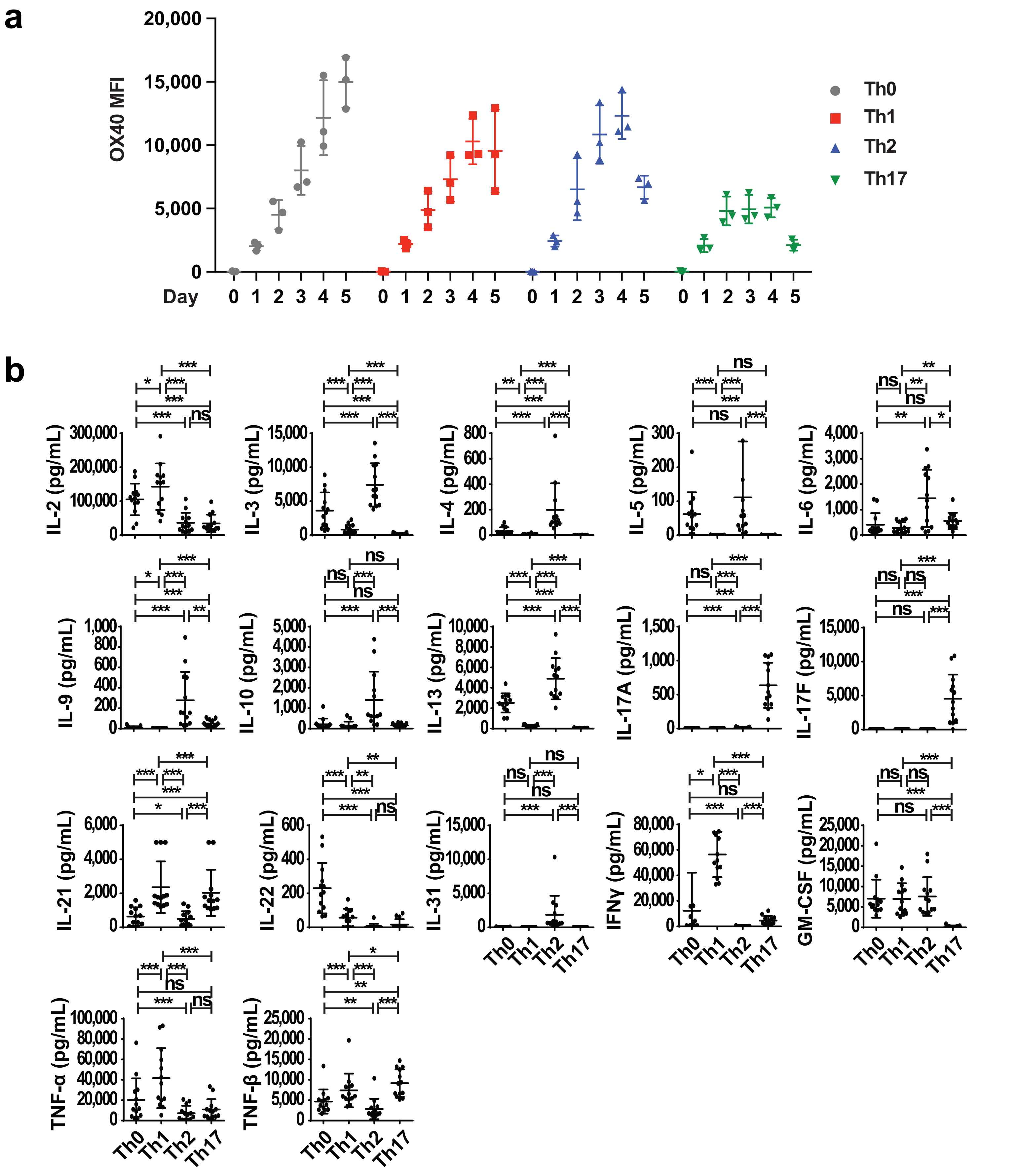

Supplementary Fig. S1: Control Th cell profiles. a) Quantification of OX40 MFI on Th0, Th1, Th2 and Th17 at Day 0 (D0), 1, 2, 3, 4 and 5 of culture. Mean ± SD and individual values from 3 donors are represented. b) Output cytokine production in each of the four Th contexts. Mean ± SD and individual values from 13 independent donors are represented. Two-sided paired Wilcoxon's test was used for statistical analysis. Statistical significance was annotated as follows: ns P >0.05; * P ≤0.05; ** P ≤0.01; *** P ≤0.001.

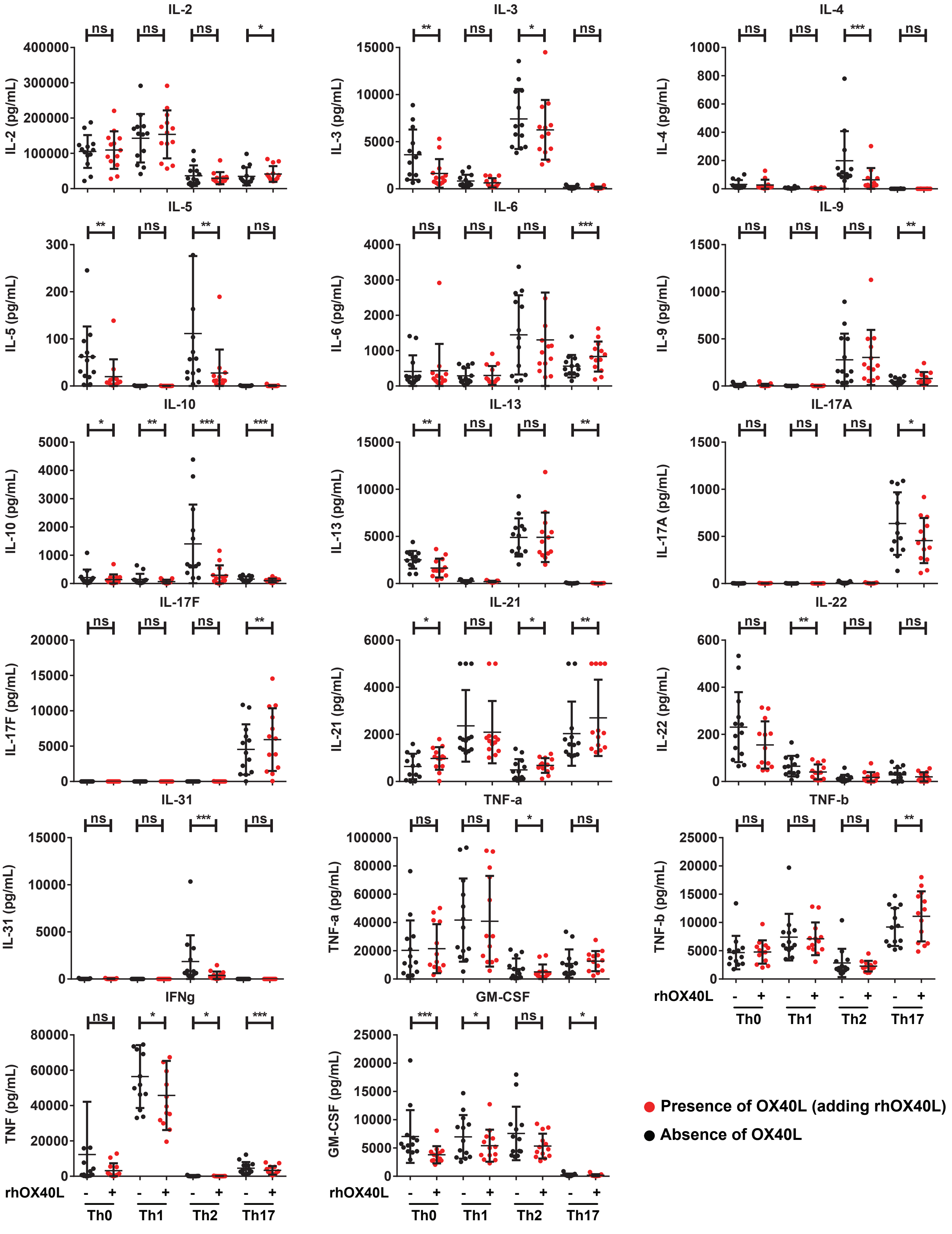

Supplementary Fig. S2: OX40L impact on output cytokine production in Th contexts. Output cytokine concentrations in presence or absence of rhOX40L, in each of the four Th contexts. Mean ± SD and individual values from 13 independent donors are represented. Two-sided paired Wilcoxon's test was used for statistical analysis. Statistical significance was annotated as follows: ns P >0.05; * P ≤0.05; ** P ≤0.01; *** P ≤0.001.

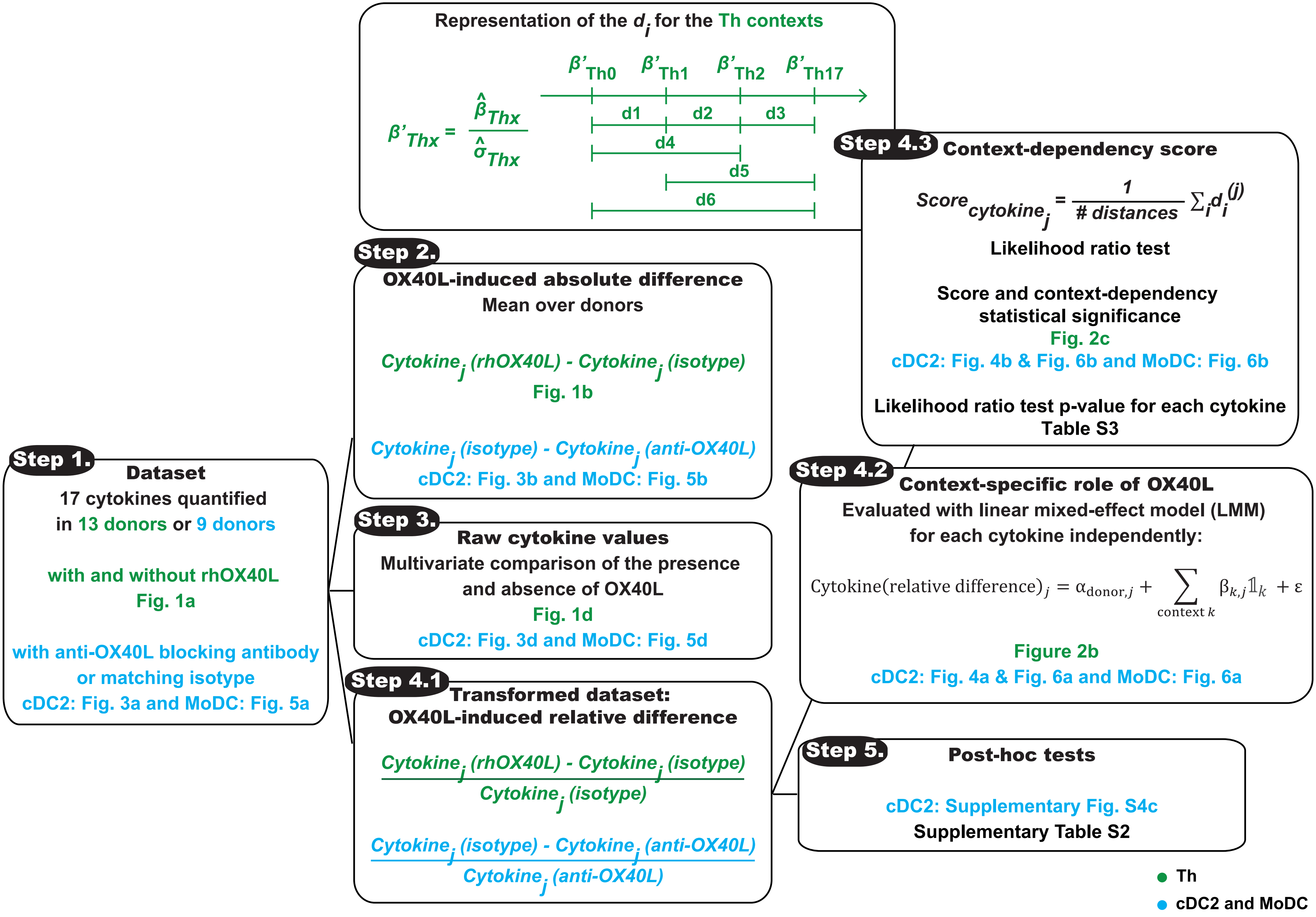

Supplementary Fig. S3: Detailed analysis strategy. Detailed mathematical modeling strategy used for data analysis and context-dependency scoring.

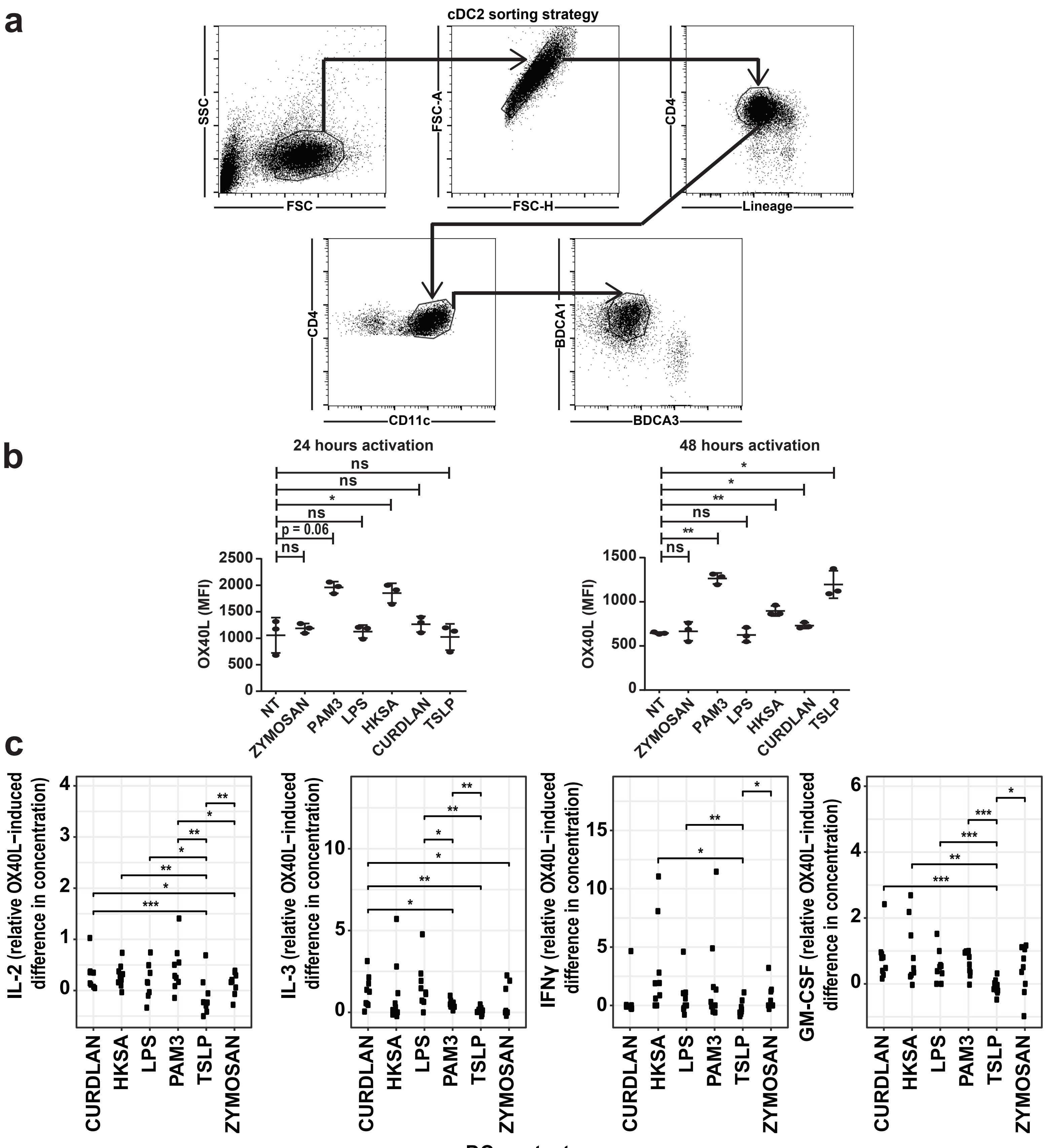

Supplementary Fig. S4: OX40L expression on cDC2. a) cDC2 sorting strategy after blood DC purification from PBMCs. b) Quantification of OX40L MFI on cDC2 activated for 24 or 48 hours without stimulation (NT), or with zymosan, PAM3, LPS, HKSA, Curdlan or TSLP. Mean ± SD and individual values from 3 donors are represented. Paired student's test was used for statistical analysis. Statistical significance was annotated as follows: ns P >0.05; * P ≤0.05; ** P ≤0.01. c) Difference in OX40L-induced effect between pairs of contexts, evaluated by likelihood ratio tests, between the linear mixed models with and without taking into account the contexts. This analysis is associated to the context-dependency scores represented in Fig. 4b. 9 independent donors were used for the analysis. Statistical significance was annotated as follows: * P ≤0.05; ** P ≤0.01; *** P ≤0.001. Only P≤0.05 are displayed.

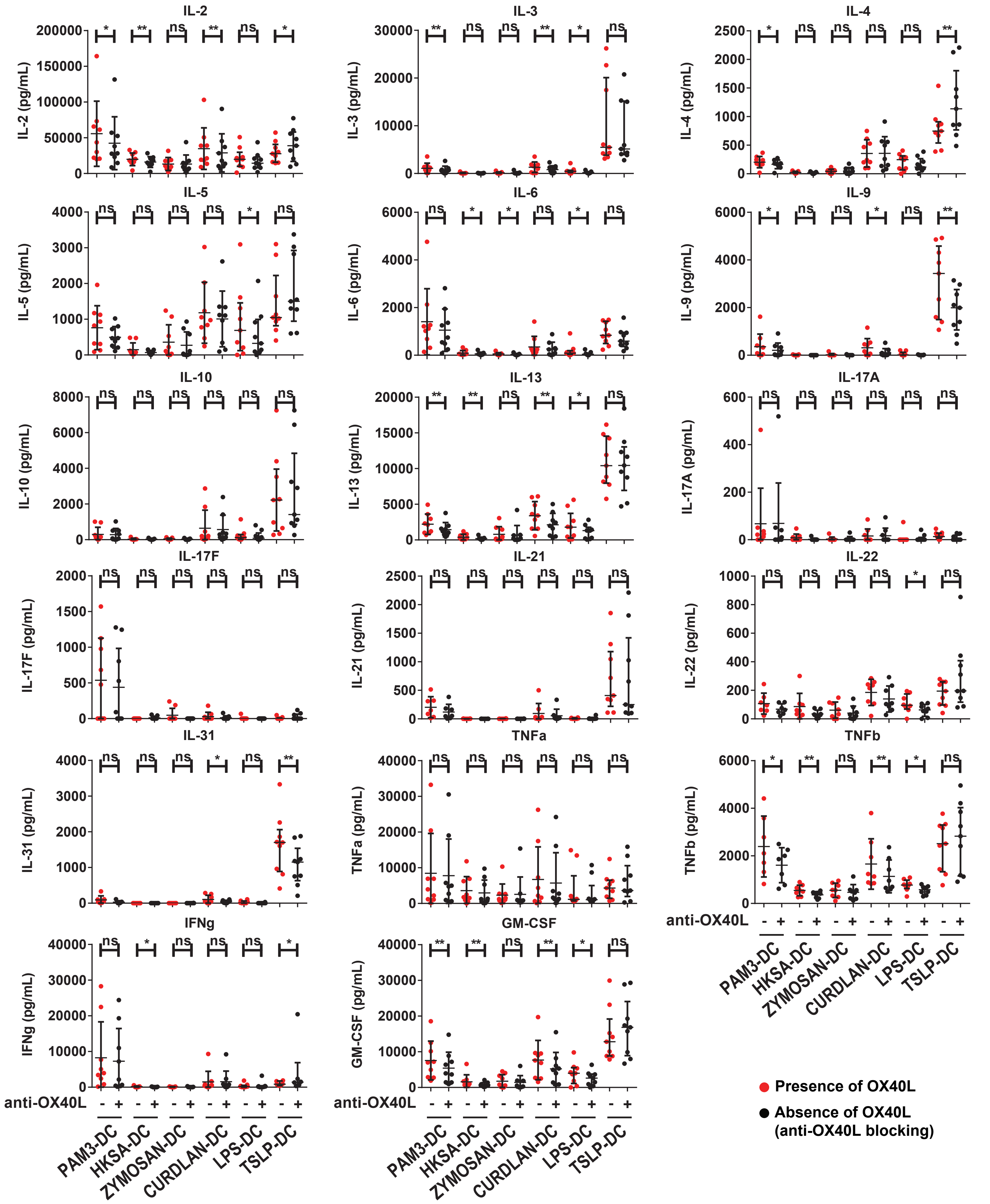

Supplementary Fig. S5: OX40L impact on output cytokine production in cDC2 contexts. Output cytokine concentrations in presence or absence of rhOX40L, in each of the six cDC2 contexts. Mean ± SD and individual values from 9 independent donors are represented. Two-sided paired Wilcoxon's test was used for statistical analysis. Statistical significance was annotated as follows: ns P >0.05; * P ≤0.05; ** P ≤0.01; *** P ≤0.001.

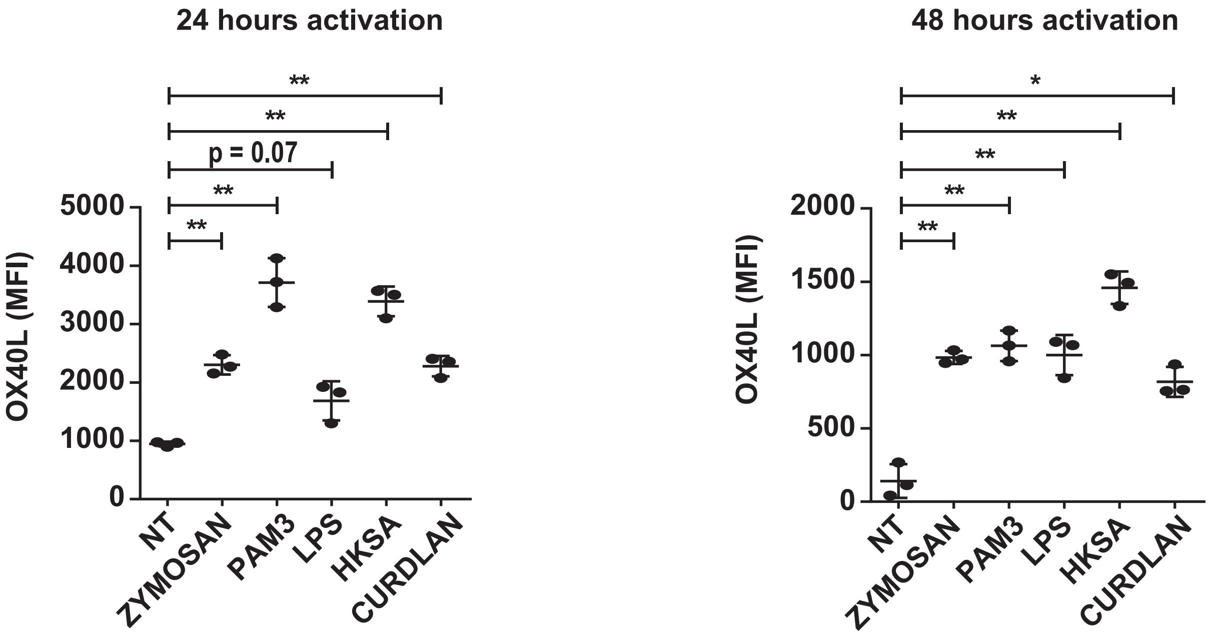

Supplementary Fig. S6: OX40L expression on MoDC. Quantification of OX40L MFI on MoDC activated for 24 or 48 hours without stimulation (NT), or with zymosan, PAM3, LPS, HKSA or Curdlan. Mean ± SD and individual values from 3 donors are represented. Paired student's test was used for statistical analysis. Statistical significance was annotated as follows: * P ≤0.05; ** P ≤0.01.

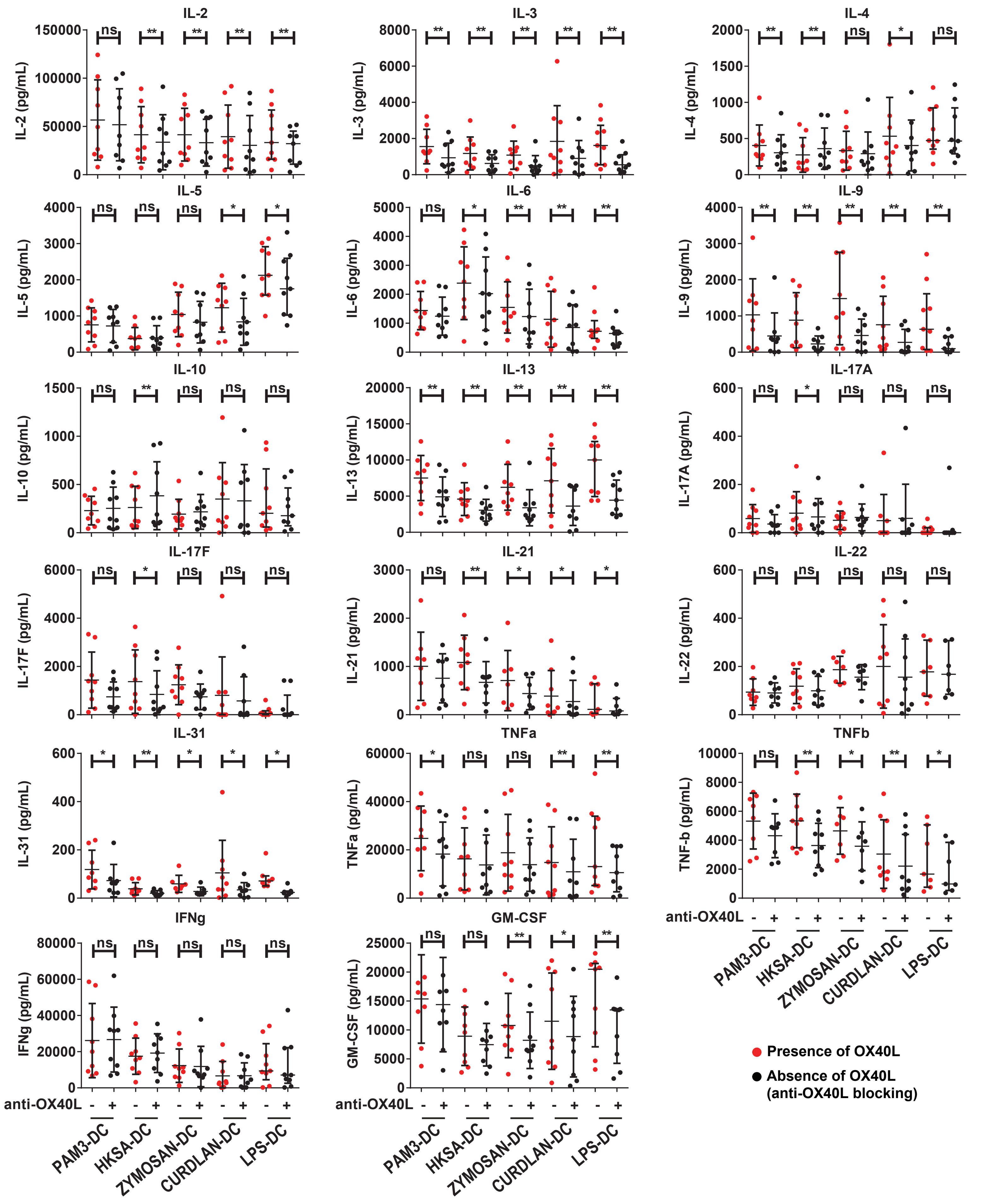

Supplementary Fig. S7: OX40L impact on output cytokine production in MoDC contexts. Output cytokine concentrations in presence or absence of rhOX40L, in each of the five MoDC contexts. Mean ± SD and individual values from 9 independent donors are represented. Two-sided paired Wilcoxon's test was used for statistical analysis. Statistical significance was annotated as follows: ns P >0.05; * P ≤0.05; ** P ≤0.01; *** P ≤0.001.
